# What to expect and when to expect it: an fMRI study of expectancy in children with ADHD symptoms

**DOI:** 10.1007/s00787-016-0921-7

**Published:** 2016-12-01

**Authors:** Branko M. van Hulst, Patrick de Zeeuw, Yvonne Rijks, Sebastiaan F. W. Neggers, Sarah Durston

**Affiliations:** NICHE Lab, Department of Psychiatry, Brain Center Rudolf Magnus, University Medical Center Utrecht, Utrecht, The Netherlands

**Keywords:** ADHD, Children, Expectancy, Timing, Inhibition, fMRI

## Abstract

Changes in cognitive control and timing have both been implicated in ADHD. Both are involved in building and monitoring expectations about the environment, and altering behavior if those expectations are violated. In ADHD, problems with expectations about future events have high face validity, as this would be associated with behavior that is inappropriate only given a certain context, similar to symptoms of the disorder. In this fMRI study, we used a timing manipulated go/nogo task to assess brain activity related to expectations about what (cognitive control) and when (timing) events would occur. We hypothesized that problems in building expectations about the environment are a more general, trans-diagnostic characteristic of children with hyperactive, impulsive and inattentive symptoms. To address this, we included children with ASD and symptoms of ADHD, in addition to children with ADHD and typically developing children. We found between-group differences in brain activity related to expectations about when (timing), but not what events will occur (cognitive control). Specifically, we found timing-related hypo-activity that was in part unique to children with a primary diagnosis of ADHD (left pallidum) and in part shared by children with similar levels of ADHD symptoms and a primary diagnosis of ASD (left subthalamic nucleus). Moreover, we found poorer task performance related to timing, but only in children with ASD and symptoms of ADHD. Ultimately, such neurobiological changes in children with ADHD symptoms may relate to a failure to build or monitor expectations and thereby hinder the efficiency of their interaction with the environment.

**Electronic supplementary material:**

The online version of this article (doi:10.1007/s00787-016-0921-7) contains supplementary material, which is available to authorized users.

## Introduction

The observation that the behavior of children with attention-deficit/hyperactivity disorder (ADHD) is inappropriate only in certain contexts has led to suggestions that children with ADHD may be particularly impaired in their ability to build expectations about future events [1]. Indeed both cognitive control, associated with expecting what events will occur, and timing, associated with expecting when they will occur, are involved in ADHD [2, 3]. However, it is unclear if these psychological processes are specific to the diagnosis of ADHD or whether they are related to ADHD symptoms in a more general, trans-diagnostic way. In this study, we analyzed cognitive control and timing in concert, using a single child-friendly fMRI paradigm in children with ASD and symptoms of ADHD, children with ADHD and typically developing children.

Nigg and Casey integrated both these neuropsychological characteristics by proposing that ADHD may be characterized by a deficient ability to build expectations about what (cognitive control) and when (timing) events will happen [1]. This ties in with theories of attention that propose that predictive models about the environment, and related anticipatory brain activity, form the foundation of attention processes [4]. From a clinical perspective, such a failure to build or monitor contextual expectations could explain behavior that is inappropriate only given a certain context, as is seen in ADHD [1], for example when a child with ADHD has trouble sitting still in the class room. From a neurobiological perspective, this theory could have a cellular basis in an existing model of reduced anticipatory dopamine signaling in ADHD [5, 6].

Cognitive control is defined as ‘the ability to override an inappropriate response in favor of another, appropriate one’ [7] and encompasses a variety of closely related constructs such as behavioral inhibition, response inhibition and inhibitory control [8]. Deficits in cognitive control have been suggested to be one of multiple, partially separable pathways to ADHD [9–12]. Neuropsychological studies of ADHD have shown deficits in cognitive control with relatively high consistency [13, 14], although it has also been estimated that at least 50% of children with ADHD do not show any deficits in this ability [9]. Moreover, neuroimaging studies have found evidence of hypo-activity in a fronto-striatal network involved in cognitive control at the group level [15–17].

Analogous to cognitive control, timing is an overarching term that encompasses different aspects of temporal processing. A wide variety of timing-related constructs have been assessed in ADHD [18] and both theoretical and empirical papers have implicated timing as a candidate neuropsychological pathway to ADHD symptoms [2, 19–21]. Moreover, a meta-analysis showed deficits in timing across multiple domains (e.g., motor timing, perceptual timing) and timescales (e.g., sub-second, supra-second timescale) in ADHD [22].

Neuropsychological changes found in children with ADHD are not necessarily specific to the diagnosis [23] and deficits in cognitive control and timing have been reported across multiple disorders [3, 24, 25]. We assessed ADHD symptoms in a trans-diagnostic way by including a third group of participants: children with a similar level of parent-rated ADHD symptoms, but a different primary diagnosis [autism spectrum disorder (ASD)]. Using a timing manipulated go/nogo task, we hypothesized that we would find changes in brain activity in fronto-striatal networks involved in motor control. We expected to find these changes in children with symptoms of ADHD, irrespective of their primary diagnosis (i.e., trans-diagnostically) and that they would be related to unexpected stimulus type and unexpected stimulus timing. Moreover, we expected that changes in brain activity would correlate more with trait ADHD symptoms than diagnosis per se. In addition, we expected that an impaired ability to build expectations in children with ADHD symptoms, would translate to less benefit from the expected timing of trials. This in turn should then translate to less speeding of response times on expected trials, compared to response times on unexpected trials (i.e. less response time benefit), as has been previously shown [26, 27].

## Methods

### Participants

A total of 108 right-handed boys, aged 8–12 years were included in the study: 33 typically developing boys and 75 boys with ADHD symptoms. Children with ADHD symptoms were recruited in two groups: 38 with a primary diagnosis of ADHD (all presentations) and 37 with a primary autism spectrum disorder (ASD) diagnosis. Typically developing children were recruited through schools in the wider Utrecht area. Children with ADHD symptoms were recruited through schools for special education and the University Medical Center Utrecht (UMCU) outpatient clinic for developmental disorders. Only children using no medication or short-acting psychostimulants (e.g., methylphenidate) were included; all parents were instructed not to administer medication in the 24 h prior to testing. All children completed a timing manipulated go/nogo paradigm [26]. After screening data quality, 29 participants were excluded on the basis of excessive head motion and three participants were excluded due to anatomical abnormalities (for details see Online Resource 1, supplementary text S1). High quality data from 76 participants were available for final analyses. Participants were matched at the group level for age and IQ. Demographics are provided in Table 1.

**Table 1:** Demographics per group

	Control (SD)	ADHD (SD)	ASD (SD)	*F* value	*p* value
N (76)	26	24	26		
Age	10.5 (1.0)	11.2 (1.1)	10.8 (1.4)	(2,73) 1.76	0.179
IQ	117.3 (18.5)	105.6 (15.9)	109.6 (17.7)	(2,72) 2.92	0.060
SWAN-hyp	0.43 (0.75)	−1.07 (0.63)**	−1.01 (0.61)**	(2,70) 40.63	<0.001*
SWAN-att	0.40 (0.63)	−1.22 (0.64)**	−1.50 (0.46)**	(2,70) 79.48	<0.001*

*ADHD* attention-deficit/hyperactivity disorder, *ASD* autism spectrum disorder, *SD* standard deviation, *SWAN-hyp* strengths and weaknesses of ADHD and normal behavior hyperactivity/impulsivity subscale, *SWAN-att* strengths and weaknesses of ADHD and normal behavior inattention subscale

* Significant overall group difference. ** Significant post hoc group difference from controls, for post hoc statistics see “Results” section

### In- and exclusion criteria

Inclusion criteria for typically developing children were: no psychiatric diagnoses on the Diagnostic Interview Schedule for Children, Fourth Edition (DISC-IV), interview [28] (with an exception of specific phobia and enuresis) and no scores in the clinical range on any scale of the Child Behavior Checklist (CBCL) [29], as reported by one of the parents. Inclusion criteria for children with ADHD symptoms were either a clinical DSM-IV diagnosis of ADHD, which was confirmed using the DISC-IV, or a clinical DSM-IV ASD diagnosis and a score in the (sub)clinical range of the CBCL subscale of Attention Problems. All clinical diagnoses were given by expert child and adolescent psychiatrists, ASD diagnoses were not otherwise confirmed. For all participants, major illness, present or past neurological illness, IQ below 70 and presence of metal objects in or around the body that would preclude MRI, were grounds for exclusion.

### Questionnaires

Parents completed the Strengths and Weaknesses of ADHD and Normal Behavior (SWAN) questionnaire [30]. This questionnaire assesses symptoms listed in the DSM-IV definition of ADHD across the complete spectrum of functioning (behavior that is below and behavior that is above that of typically developing peers is quantified).

### Timing manipulated go/nogo paradigm

We used a timing manipulated go/nogo task [26]. Participants were instructed to help a mouse collect cheese. In a majority of trials (82%), a picture of a door was shown for 3500 ms (resulting in expected timing of the ensuing stimulus), in a minority of trials (18%) the door was shown for 1500 ms (resulting in unexpected timing of the ensuing stimulus). Subsequently, either a piece of cheese (go trials; 82% of 264 trials) or a cat (nogo trials; 18% of 264 trials) was shown for 500 ms. Subjects were asked to press a button as fast as possible when a piece of cheese was shown and to withhold their response when a cat was shown. These two manipulations created four types of trials: go trials with expected timing, go trials with unexpected timing, nogo trials with expected timing and nogo trials with unexpected timing. Participants included in this study performed two different tasks in a single scan session, interrupted by a short (15-min) break. Results from the other task (a child-friendly monetary incentive delay paradigm) were analyzed separately (van Hulst et al., submitted for publication).

### fMRI acquisition

The study was run on a 3.0-T Achieva MRI scanner (Philips Medical Systems, Best, the Netherlands) using an eight-channel sensitivity-encoding (SENSE) parallel imaging head coil. For anatomical reference, we acquired a whole-brain three-dimensional fast field echo T1-weighted scan (200 slices; repetition time = 10 ms; echo time = 4.6 ms; flip angle = 8°; field of view, 240 × 240 × 160 mm; voxel size: 0.75 × 0.8 × 0.75 mm isotropic). In addition, we acquired whole-brain T2*-weighted echo planar images (EPI) with blood-oxygen level-dependent (BOLD) contrast (4 sessions; 135 volumes per session; 36 slices per volume; interleaved acquisition; TR = 2.02 s; TE = 35 ms; field of view = 232 × 123 × 256 mm; flip angle = 70°; voxel size = 2.67 × 2.67 × 3.43 mm) oriented in a transverse plane. We collected six dummy scans to allow for T1 equilibration effects.

### Preprocessing of fMRI data

We analyzed FMRI data using SPM8 (r4290) (http://www.fil.ion.ucl.ac.uk/spm/software/spm8) as implemented in Matlab 7.12 (Mathworks Inc., Natick, MA, USA). To correct for between-scan head motion, we realigned all images to the first volume using rigid body transformations. Next, the anatomical image was co-registered to the first fMRI image using the mutual information criteria method and subsequently normalized to Montreal Neurological Institute (MNI) space using unified segmentation. We then resliced the image at a voxel size of 1.0 × 1.0 × 1.0 mm. Functional images were normalized using the normalization parameters generated in this step, the images were resliced at a voxel size of 3.0 × 3.0 × 3.0 mm. Finally, we spatially smoothed the fMRI images using a Gaussian kernel with a full width at half maximum (FWHM) of 6 mm. In addition, we assessed scan-to-scan movement using ArtRepair [31]. Scans with more than 1.0 mm scan-to-scan movement or more than 1.5% deviation from the average global signal, were replaced using a linear interpolation of the values of neighboring scans. Participants with more than 30% corrected scans were excluded from further analyses (for details, see Online Resource 1, supplementary text S1).

### Statistical analyses—task performance

We tested for an effect of diagnosis on five measures of task performance: baseline mean response times (MRT_expected-go_), baseline standard deviation of response times (SDRT_expected-go_); percentage of correct go trials (accuracy_go_), percentage of correct nogo trials (accuracy_nogo_) and response time benefit (RT_benefit_). RT_benefit_ denotes the difference in MRT between expected and unexpected go trials. This difference is expressed in the number of standard deviations of MRT_expected-go_ (MRT_expected-go_ − MRT_unexpected-go_)/SDRT_expected-go_. After testing for normality (Shapiro–Wilk test) and homogeneity of variances (Levene’s test), analysis of covariance (ANCOVA) was conducted with diagnosis as factor. If age or IQ significantly covaried with a measure of task performance, analyses were run, reported and interpreted both with and without the covariate for additional insight. If they did not, the covariates were left out of the final model. Where group differences were found, we performed post hoc testing using Fisher’s least significant difference (LSD). Moreover, we followed up with dimensional analyses by testing for an effect of attention problems (i.e., SWAN-inattention subscores) or hyperactivity/impulsivity (i.e., SWAN-hyperactivity/impulsivity subscores) on these performance measures, within the combined clinical group and the control group separately. Where no group differences were found, correlational analyses were run on the entire sample. Results were corrected for multiple comparisons using False Discovery Rate (FDR) correction on the separate ANCOVA results (per task performance measure) using the Benjamini-Hoghberg method [32].

### Statistical analyses—fMRI

Statistical analyses of fMRI data were performed in a two-level procedure within the framework of the general linear model. First, for each subject, we modeled the blood oxygenation level dependent (BOLD) activation invoked by task cues as conditions of interest, and realignment parameters as potential confounders (condition of no interest). We modeled cue onset of four different cues as conditions of interest: expected go trials, unexpected go trials, expected nogo trials and unexpected nogo trials. Regressors were created by convolving delta functions coding for cue onset with a canonical hemodynamic response function (as implemented in SPM8) for each cueing category separately. The estimated regression coefficients for the different cues were then contrasted, resulting in two first-level contrast images for each subject: go versus nogo trials and expected versus unexpected trials. Data were high-pass filtered using discrete cosine basis functions with a 128-s cut-off. The analysis focused on average activity in a priori specified regions of interests (ROIs). We used regions that are considered part of fronto-striato-cerebellar loops involved in motor control and, more specifically, in response inhibition and temporal processing [17, 22]. ROIs were created using different atlases provided in the FSL software package (the Harvard-Oxford cortical and subcortical structural atlases, the Probabilistic cerebellar atlas and the Subthalamic nucleus atlas). Six fronto-striatal regions putatively involved in response inhibition or motor timing were selected per hemisphere: anterior cingulate, inferior frontal gyrus, putamen, pallidum, supramarginal gyrus and subthalamic nucleus. In addition, a cerebellar vermis ROI was created, adding up to a total of 13 ROIs. If results were found in a given ROI, a supplementary figure of the map is provided, see Online Resource 1, supplementary Figures S1 and S2. Average activity per ROI was operationalized as average *β* values of the contrast image and extracted using Marsbar (http://marsbar.sourceforge.net/). Main effects of expectancy and inhibition were analyzed in typically developing children using a single-factor analysis of variance (ANOVA). For all ROIs, we performed a two-factor repeated measures ANOVA using activity related to the four different conditions of interest separately to test for two- and three-way interactions between group status, expectancy and inhibition. To test for group differences in brain activity we conducted an ANCOVA per ROI with diagnosis as factor. Where age and IQ significantly covaried with activity in an ROI, analyses were run, reported and interpreted both with and without the covariate for- additional insight. If not, the covariates were left out of the final model. Where group differences were found we performed post hoc testing using Fisher’s least significant difference (LSD) to compare the three groups. In a follow-up analysis, nogo accuracy and RT_benefit_ were included as an additional performance-related covariate. In further follow-up analyses, we tested for effects of ADHD symptoms (i.e., scores on two separate SWAN subscales) on brain activity (using ANCOVA) within the combined clinical group and control group separately. If group differences were not found, correlational analyses were run on the entire sample. Results were corrected for multiple comparisons using False Discovery Rate (FDR) correction on the separate ANCOVA results (per ROI) using the Benjamini-Hoghberg method [32]. In addition, we ran exploratory, post hoc, whole-brain analyses (see Online Resource 1: Supplementary Text S2, Supplementary Table S4 and S5).

## Results

### Questionnaires

ANOVA showed group differences on the inattention scale of the SWAN and the hyperactivity/impulsivity scale of the SWAN (see Table 1). Post hoc testing indicated that both clinical groups had lower scores on the two SWAN subscales than typically developing children, corresponding to more symptoms of ADHD. No differences were found between children with ADHD and children with ASD and ADHD symptoms.

### Task performance

ANOVA showed between-group differences on mean response times (MRT_expectedgo_), standard deviation of response times (SDRT_expectedgo_), percentage of correct go trials (Accuracy_go_) and response time benefit (RT_benefit_) (see Table 2). No effects of age or IQ were found, and accordingly, we left those out of the final model. As SDRT_expectedgo_ and Accuracy_go_ were not normally distributed, we confirmed these results using a Kruskal–Wallis test (SDRT_expectedgo_: H(2) = 13.23, *p* = 0.001; Accuracy_go_: H(2) = 8.73, *p* = 0.13). Post hoc testing showed that compared to typically developing children, both clinical groups had longer response times (high MRT_expectedgo_); higher variability of response times (SDRT_expectedgo_) and lower go-trial accuracy (Accuracy_go_). Furthermore, children with combined ASD and ADHD symptoms benefitted less from trials with expected timing than controls and children with ADHD (lower RT_benefit_).

**Table 2:** Task performance measures per group

Variable	Control (SD)	ADHD (SD)	ASD (SD)	*F* value	*p* value
MRT_expgo_	433.0 (38.0)	471.3 (48.1)**	470.0 (55.1)**	(2,73) 5.37	0.007*
SDRT_expgo_	96.9 (23.0)	128.6 (44.3)**	122.9 (47.7)**	(2,73) 4.61	0.013*
Go_accuracy_	94.4 (6.5)	86.2 (11.3)**	89.1 (9.6)**	(2,73) 5.04	0.009*
Nogo_accuracy_	48.8 (15.1)	52.7 (16.4)	45.7 (18.1)	(2,73) 1.13	0.329
RT_benefit_	0.84 (0.36)	0.77 (0.40)	0.51 (0.31)**	(2,73) 5.94	0.004*

*ADHD* attention-deficit/hyperactivity disorder, *ASD* autism spectrum disorder, *SD* standard deviation, *MRT*_*expgo*_ mean response time on go trials with expected timing, *SDRT*_*expgo*_ standard deviation of response times on go trials with expected timing, *Go*_*accuracy*_ percentage of correct go trials, *Nogo*_*accuracy*_ percentage of correct nogo trials, *RT*_*benefit*_ response time benefit

* Significant group difference. ** Significant post hoc group difference from controls, for post hoc statistics see “Results” section

### Brain activity

Typically developing children showed a main effect of temporal expectancy in all ROIs except cerebellar vermis and right inferior frontal gyrus, (for details of main effects see Online Resource 1, supplementary Table S1a and S1b). A main effect of stimulus type was found in bilateral supramarginal gyrus, bilateral anterior cingulate, bilateral vermis and right pallidum. We found an inhibition by expectancy interaction for activity in bilateral pallidum and putamen (left pallidum: F(1,73) = 5.07, *p* = 0.027; right pallidum: F(1,73) = 10.65, *p* = 0.002; left putamen: F(1,73) = 8.41, *p* = 0.005; right putamen: F(1,73) = 7.83, *p* = 0.007). We found no three-way (inhibition by expectancy by group) interactions for activity in any of the ROIs.

**Fig. 1:**
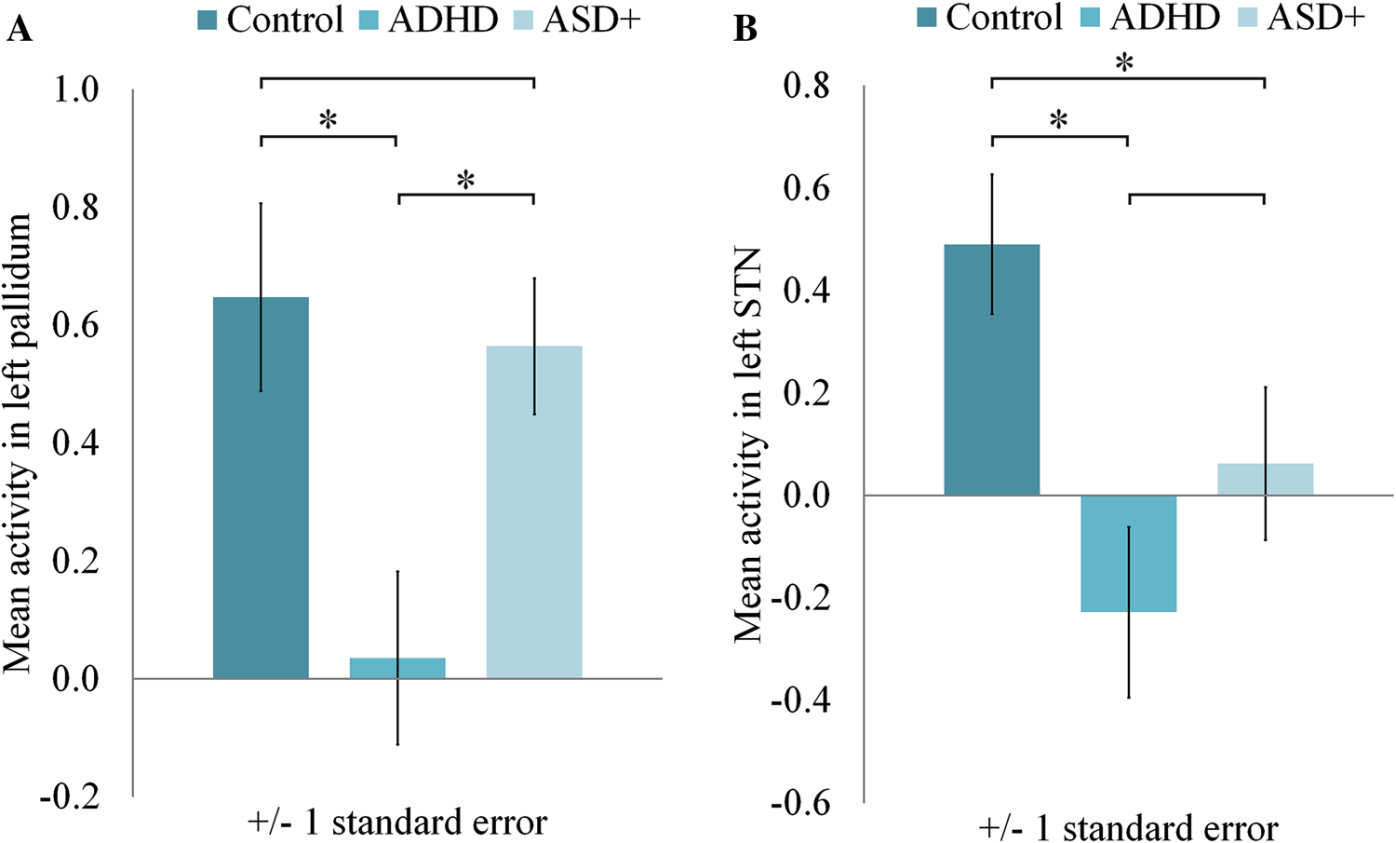
Timing-related brain activity in left pallidum and left subthalamic nucleus. *STN* subthalamic nucleus; *ADHD* attention-deficit/hyperactivity disorder, *ASD* ^*+*^ autism spectrum disorder and symptoms of ADHD. Timing-related brain activity per group is shown for two regions of interest: left pallidum (**a**) and left subthalamic nucleus (**b**). For both regions of interest, ANOVA results were significant after FDR correction for multiple comparisons. *Significant post hoc group difference

We found between-group differences in brain activation related to temporal expectancy in left STN (F(2,73) = 5.72, *p* = 0.005) and left pallidum (F(2,73) = 5.36, *p* = 0.007) (see Fig. 1, for activation in all ROIs see Online Resource 1, supplementary Table S2a and S2b). We found no effects of age or IQ and accordingly they were left out of the final model. Post hoc analyses showed that in left STN, both clinical groups (ADHD: *M* = −0.23, SD = 0.81; ASD: *M* = 0.06, SD = 0.76) had less activation than typically developing children (*M* = 0.49, SD = 0.69). Notably, in left pallidum, we found a dissociation between the clinical groups, with children with ADHD showing less activation (*M* = 0.04, SD = 0.72) than both typically developing children (*M* = 0.65, SD = 0.81) and children with ASD and ADHD symptoms (*M* = 0.56, SD = 0.59). Because there was an interaction between inhibition and expectancy in pallidum, we also tested for an effect of expectancy in go and nogo conditions separately. The group difference in brain activity related to expectancy, only retained significance for the nogo condition, again with children with ADHD showing less activation (*M* = −0.13, SD = 0.59) than both typically developing children (*M* = 0.33, SD = 0.52) and children with ASD and ADHD symptoms (*M* = 0.18, SD = 0.50).

We found no associations between any of the questionnaire measures and any of the brain activity measures. In a follow-up analysis, we found that task performance (i.e. _RTbenefit_) significantly covaried with activity in left pallidum and left putamen (see Online Resource 1, supplementary Table S3). We found that with RT_benefit_ as covariate in the model, between-group differences in left STN and left pallidum retained significance and an additional between-group difference was found in left putamen (F(2,73) = 5.67, *p* = 0.005).

## Discussion

We used task-based fMRI to investigate how children with symptoms of ADHD build and monitor expectations about what events will occur (cognitive control) and *when* events will occur (timing). We found that two brain regions in children with ADHD showed hypo-activity related to the timing of events. Children with ADHD and children with ASD and ADHD symptoms both showed timing-related hypo-activity in the left subthalamic nucleus. However, timing-related hypo-activity in left pallidum was only found in children with a primary diagnosis of ADHD. Children with ASD and ADHD symptoms benefitted less in terms of their response time from trials with expected timing, than typically developing children and children with ADHD. We found no between-group differences in brain activity or task performance related to expectations about *what* events will occur.

Nigg and Casey have hypothesized that children with ADHD may show a context-dependent deficit in generating or monitoring predictions [1]. Indeed, we found that children with ADHD showed reduced brain activity related to the temporal predictability of events. This timing-related hypo-activity was found in left subthalamic nucleus and left pallidum, in line with studies that have proposed a role for the basal ganglia in timing deficits in ADHD [2, 18, 33], but contradicting meta-analytical findings [22]. However, [22] noted that a further differentiation of results in separate timing domains could prove pivotal to find specific temporal processing deficits in ADHD. Remarkably, both subthalamic nucleus and pallidum have a prominent role in the striatal beat frequency theory of interval timing in which these structures act as a coincidence detector, signaling patterns of oscillatory activity matching a previously learned time interval [24, 34]. As such, the subthalamic nucleus and pallidum are considered key areas in the temporal monitoring of predictive models. Our finding of timing-related striatal hypo-activity links the striatal beat frequency theory [34] with theories on dopamine signaling in ADHD [e.g. 5], as in both theories reduced striatal dopamine signaling leads to reduced anticipatory brain activity.

We hypothesized that any deficits found would not be specific to children with ADHD, but would be apparent in both groups of children with ADHD symptoms. Indeed, we found subthalamic hypo-activity in children with ASD and ADHD symptoms and in children with ADHD. However, hypo-activity in left putamen was only found in children with a primary diagnosis of ADHD. We did not expect to find such a clear categorical difference in brain activity between two groups with similar levels of ADHD symptoms. Notably, this finding is in line with reasoning that was made explicit in DSM-IV, where it states that, although symptoms of inattention, hyperactivity and impulsivity may appear alike in children with ADHD and children with ASD, they stem from different neuropsychological problems and should thus be approached differentially. In addition, similar hypo-activity in subthalamic nucleus in children with ADHD and in children with ASD and ADHD symptoms could not be explained in a trans-diagnostic way (i.e., by ADHD symptoms alone) and was not reflected in task performance. This suggests that the mechanisms underlying subthalamic hypo-activity might differ between children with ASD and children with ADHD. Deficient temporal processing in ASD has been found in several studies [e.g. 35, 36] and has been related to atypical sensory processing [37, 38]. Reduced benefit from expected timing of trials could be related to enhanced sensory processing of less relevant stimuli [39]. This way, in children with ASD, cues with expected and unexpected timing may be judged equally relevant and processed equally fast.

We did not find differences in inhibitory performance or brain activity related to the ‘what’ component of expectancy in children with ADHD symptoms (i.e., cognitive control). This is unexpected, as several meta-analyses have reported hypo-activity during response inhibition tasks in children with ADHD [15–17]. Our task had a relatively low event rate (four second inter-trial interval), and as such it is possible that it overtaxed attentional control, especially in this age-range. This explanation is supported by lower go accuracy (86%) and slower response times (471 ms) for children with ADHD than for typically developing children (respectively, 94%, 433 ms). Both metrics have been linked to attentional lapses [40, 41] and could explain why we did not find differences in cognitive control.

Problems in building and monitoring expectations about future environmental events have high face validity for children with ADHD symptoms, as they would be expected to lead to behavior that is inappropriate only given a certain context, similar to what is seen in the disorder. An example of this contextual deficit is the class room environment, in which simple actions such as moving and talking are allowed only under strict (temporal and spatial) conditions. If neurobiological changes in children with ADHD symptoms indeed relate to problems with building or monitoring predictive models, this could provide an impulse for behavioral interventions to directly target expectation building or predictability.

### Limitations

In this study, we focused on thirteen a priori regions of interest. In the cerebellum, we chose to analyze only the cerebellar vermis, as meta-analytical studies have found this region to be smaller [42] and less active [22] in individuals with ADHD. However, previous studies using a similar task design reported decreased activity in inferior cerebellum related to stimulus timing [26, 27]. This region was not fully included in our field of view and accordingly we cannot make any (post hoc) statements about this region.

## Electronic supplementary material

Below is the link to the electronic supplementary material.

Supplementary material 1 (DOCX 2124 kb)

## References

[1] Nigg JT, Casey BJ (2005). An integrative theory of attention-deficit/hyperactivity disorder based on the cognitive and affective neurosciences. Dev Psychopathol.

[2] Noreika V, Falter CM, Rubia K (2013). Timing deficits in attention-deficit/hyperactivity disorder (ADHD): evidence from neurocognitive and neuroimaging studies. Neuropsychologia.

[3] Lipszyc J, Schachar R (2010). Inhibitory control and psychopathology: a meta-analysis of studies using the stop signal task. J Int Neuropsychol Soc.

[4] Ghajar J, Ivry RB (2009). The predictive brain state: asynchrony in disorders of attention?. Neuroscientist.

[5] Tripp G, Wickens JR (2008). Research review: dopamine transfer deficit: a neurobiological theory of altered reinforcement mechanisms in ADHD. J Child Psychol Psychiatry.

[6] Tripp G, Wickens JR (2009). Neurobiology of ADHD. Neuropharmacology.

[7] Casey BJ, Tottenham N, Liston C, Durston S (2005). Imaging the developing brain: what have we learned about cognitive development?. Trends Cogn Sci.

[8] Aron AR (2007). The neural basis of inhibition in cognitive control. Neurosci.

[9] Nigg JT, Willcutt EG, Doyle AE, Sonuga-Barke EJS (2005). Causal heterogeneity in attention-deficit/hyperactivity disorder: do we need neuropsychologically impaired subtypes?. Biol Psychiatry.

[10] Solanto MV, Abikoff H, Sonuga-Barke E (2001). The ecological validity of delay aversion and response inhibition as measures of impulsivity in AD/HD: a supplement to the NIMH multimodal treatment study of AD/HD. J Abnorm Child Psychol.

[11] Sonuga-Barke EJS (2002). Psychological heterogeneity in AD/HD–a dual pathway model of behaviour and cognition. Behav Brain Res.

[12] Sonuga-Barke EJS (2005). Causal models of attention-deficit/hyperactivity disorder: from common simple deficits to multiple developmental pathways. Biol Psychiatry.

[13] Hervey AS, Epstein JN, Curry JF (2004). Neuropsychology of adults with attention-deficit/hyperactivity disorder: a meta-analytic review. Neuropsychology.

[14] Willcutt EG, Doyle AE, Nigg JT (2005). Validity of the executive function theory of attention-deficit/hyperactivity disorder: a meta-analytic review. Biol Psychiatry.

[15] Cortese S, Kelly C, Chabernaud C (2012). Toward systems neuroscience of ADHD: a meta-analysis of 55 fMRI studies. Am J Psychiatry.

[16] Hart H, Radua J, Nakao T (2013). Meta-analysis of functional magnetic resonance imaging studies of inhibition and attention in attention-deficit/hyperactivity disorder: exploring task-specific, stimulant medication, and age effects. JAMA Psychiatry.

[17] McCarthy H, Skokauskas N, Frodl T (2014). Identifying a consistent pattern of neural function in attention deficit hyperactivity disorder: a meta-analysis. Psychol Med.

[18] Toplak ME, Dockstader C, Tannock R (2006). Temporal information processing in ADHD: findings to date and new methods. J Neurosci Methods.

[19] De Zeeuw P, Weusten J, van Dijk S (2012). Deficits in cognitive control, timing and reward sensitivity appear to be dissociable in ADHD. PLoS One.

[20] Sonuga-Barke EJS, Bitsakou P, Thompson M (2010). Beyond the dual pathway model: evidence for the dissociation of timing, inhibitory, and delay-related impairments in attention-deficit/hyperactivity disorder. J Am Acad Child Adolesc Psychiatry.

[21] van Hulst BM, de Zeeuw P, Durston S (2015). Distinct neuropsychological profiles within ADHD: a latent class analysis of cognitive control, reward sensitivity and timing. Psychol Med.

[22] Hart H, Radua J, Mataix-Cols D, Rubia K (2012). Meta-analysis of fMRI studies of timing in attention-deficit hyperactivity disorder (ADHD). Neurosci Biobehav Rev.

[23] Rommelse NNJ, Geurts HM, Franke B (2011). A review on cognitive and brain endophenotypes that may be common in autism spectrum disorder and attention-deficit/hyperactivity disorder and facilitate the search for pleiotropic genes. Neurosci Biobehav Rev.

[24] Allman MJ, Meck WH (2012). Pathophysiological distortions in time perception and timed performance. Brain.

[25] Geurts HM, van den Bergh SFWM, Ruzzano L (2014). Prepotent response inhibition and interference control in autism spectrum disorders: two meta-analyses. Autism Res.

[26] Durston S, Davidson MC, Mulder MJ (2007). Neural and behavioral correlates of expectancy violations in attention-deficit hyperactivity disorder. J Child Psychol Psychiatry.

[27] Mulder MJ, Baeyens D, Davidson MC (2008). Familial vulnerability to ADHD affects activity in the cerebellum in addition to the prefrontal systems. J Am Acad Child Adolesc Psychiatry.

[28] Shaffer D, Fisher P, Lucas CP (2000). NIMH Diagnostic interview schedule for children version IV (NIMH DISC-IV): description, differences from previous versions, and reliability of some common diagnoses. J Am Acad Child Adolesc Psychiatry.

[29] Verhulst F, Van Der Ende J, Koot H (1996). Handleiding voor de CBCL/4–18 (manual for the CBCL/4–18). Department of child and adolescent psychiatry.

[30] Lakes KD, Swanson JM, Riggs M (2012). The reliability and validity of the english and spanish strengths and weaknesses of ADHD and normal behavior rating scales in a preschool sample: continuum measures of hyperactivity and inattention. J Atten Disord.

[31] Mazaika PK, Hoeft F, Glover GH, Reiss AL (2009). Methods and software for fMRI analysis of clinical subjects. Neuroimage.

[32] Benjamini Y, Hochberg Y, Hochberg Y (1995). Controlling the false discovery rate: a practical and powerful approach to multiple testing. J R Stat Soc B.

[33] Valera EM, Spencer RMC, Zeffiro TA (2010). Neural substrates of impaired sensorimotor timing in adult attention-deficit/hyperactivity disorder. Biol Psychiatry.

[34] Buhusi CV, Meck WH (2005). What makes us tick? Functional and neural mechanisms of interval timing. Nat Rev Neurosci.

[35] Szelag E, Kowalska J, Galkowski T, Pöppel E (2004). Temporal processing deficits in high-functioning children with autism. Br J Psychol.

[36] McPartland J, Dawson G, Webb SJ (2004). Event-related brain potentials reveal anomalies in temporal processing of faces in autism spectrum disorder. J Child Psychol Psychiatry.

[37] Stevenson RA, Segers M, Ferber S (2015). Keeping time in the brain: autism spectrum disorder and audiovisual temporal processing. Autism Res.

[38] Brock J, Brown CC, Boucher J, Rippon G (2002). The temporal binding deficit hypothesis of autism. Dev Psychopathol.

[39] Marco EJ, Hinkley LBN, Hill SS, Nagarajan SS (2011). Sensory processing in autism: a review of neurophysiologic findings. Pediatr Res.

[40] Weissman DH, Roberts KC, Visscher KM, Woldorff MG (2006). The neural bases of momentary lapses in attention. Nat Neurosci.

[41] Metin B, Roeyers H, Wiersema JR (2012). A meta-analytic study of event rate effects on Go/No-Go performance in attention-deficit/hyperactivity disorder. Biol Psychiatry.

[42] Valera EM, Faraone SV, Murray KE, Seidman LJ (2007). Meta-analysis of structural imaging findings in attention-deficit/hyperactivity disorder. Biol Psychiatry.

