# Supplementary material for: What to expect and when to expect it: an fMRI study of expectancy in children with ADHD symptoms
Source: Eur Child Adolesc Psychiatry. 2016 Dec 1;26(5):583–90. doi: 10.1007/s00787-016-0921-7 (PMC5394180; doi:10.1007/s00787-016-0921-7)
Supplement: Supplementary file 1 — Supplementary material 1 (DOCX 2124 kb) [file 787_2016_921_MOESM1_ESM.docx]

### **Online Resource 1:** Supplementary Text, Figures and Tables

### **Article:** What to expect and when to expect it: an fMRI study of expectancy in children with ADHD symptoms

**Journal:** European Child & Adolescent Psychiatry

**Authors:** Branko M. van Hulst, Patrick de Zeeuw, Yvonne Rijks, Sebastiaan F.W. Neggers, Sarah Durston

**Affiliation:** NICHE Lab, Department of Psychiatry, Brain Center Rudolf Magnus, University Medical Center Utrecht, Utrecht, The Netherlands

**Corresponding author:** Branko M. van Hulst, email: b.vanhulst@umcutrecht.nl

**Supplementary Text S1.** Screening of data quality

All T1-weighted scans were assessed by an expert radiologist. Following this step, three participants were excluded for anatomical abnormalities (in all cases, an arachnoid cyst). Scan-to-scan movement was assessed using ArtRepair (Mazaika, Hoeft, Glover, & Reiss, 2009). Scans with more than 1.0 mm scan-to-scan movement and scans with a more than 1.5% deviation from the average global signal, were replaced using a linear interpolation of the values of neighboring scans. Recordings from task levels with more than 30% corrected scans were excluded from further analyses. Subsequently, participants with less than three levels of sufficient data quality were excluded. 29 Participants (4 typically developing children, 14 children with ADHD and 11 children with ASD) were excluded on the basis of this criterion.

**Supplementary Text S2.** Exploratory post-hoc whole brain analyses

We ran whole-brain analyses for activity related to inhibition and expectancy (i.e. task effects) by conducting one-sample t-tests on the two separate contrast images of typically developing children. We corrected for multiple comparisons using a family wise error (FWE) correction and a minimum cluster size of five voxels. The results are reported in two tables (S4 and S5) included in Online Resource 1. Also we tested for whole-brain between-group differences using ANOVA with group as factor and activity in the two separate contrast images (inhibition and expectancy) as dependent variable. No brain areas showed significant between-group differences when correcting for multiple comparisons using whole brain FWE correction and a minimum cluster size of five voxels..

**Supplementary Fig. S1.** Region of interest map for left pallidum


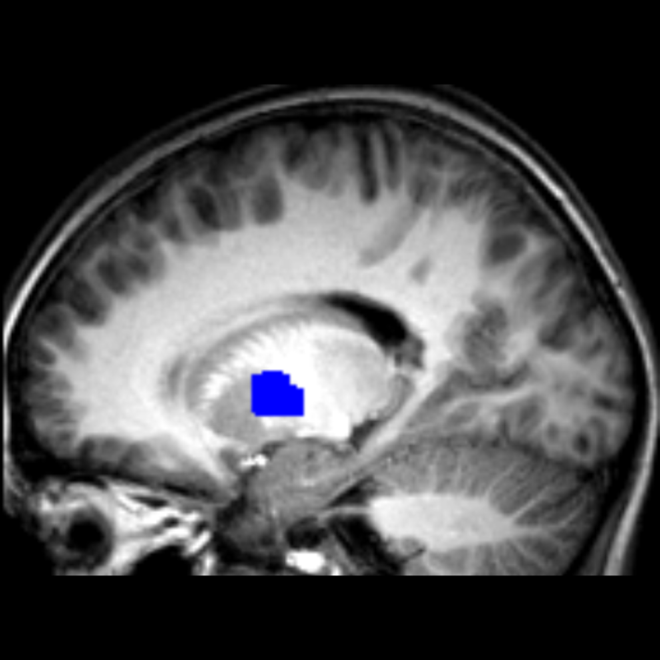

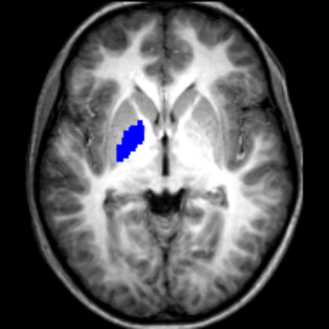

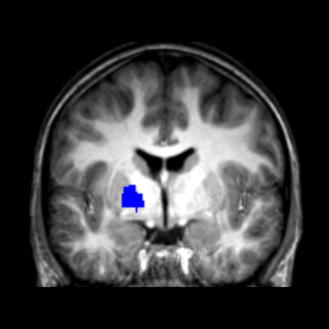


**Note.** Supplementary Figure 1 shows three views (sagittal, axial and coronal) of the atlas-based left pallidum map that was used for the region of interest analyses. The sections were placed at MNI-coordinates (x, y, z respectively): -17 -4 -3.

**Supplementary Fig. S2.** Region of interest map for left subthalamic nucleus


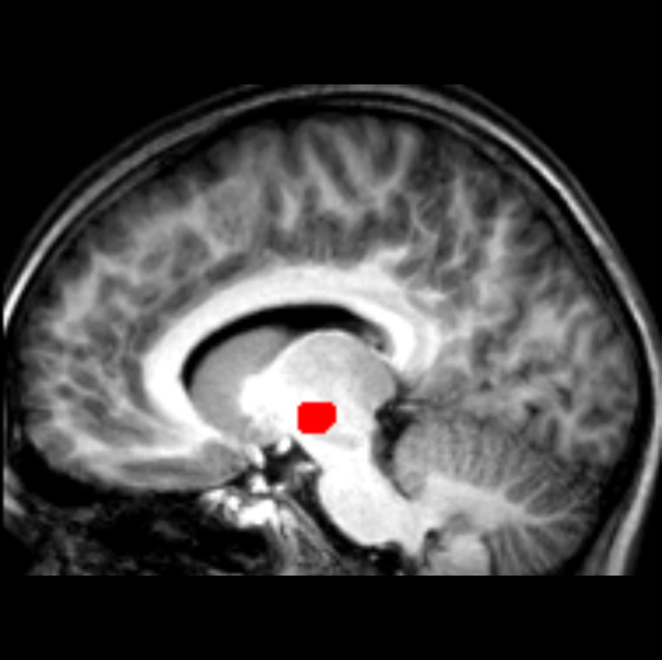

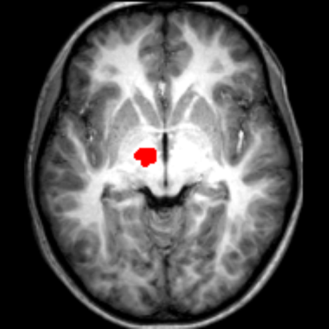

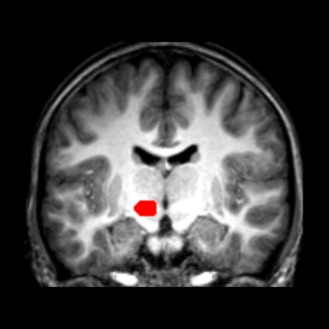


**Note.** Supplementary Figure 2 shows three views (sagittal, axial and coronal) of the atlas-based left subthalamic nucleus map that was used for the region of interest analyses. The sections were placed at MNI-coordinates (x, y, z respectively): -10 -15 -7.

**Supplementary Table S1a.** Main effect of expectancy

| ROI | Number of voxels | Control (SD) | F-value  (1,25) | p-value |
| --- | --- | --- | --- | --- |
| l-smg | 1057 | 0.92 (1.04) | 20.19 | <0.001* |
| r-smg | 1015 | 0.69 (1.51) | 5.30 | 0.030* |
| l-ifg | 705 | 1.00 (1.46) | 12.26 | 0.002* |
| r-ifg | 675 | 0.64 (1.85) | 3.13 | 0.089 |
| l-pal | 77 | 0.65 (0.81) | 16.50 | <0.001* |
| r-pal | 75 | 0.60 (0.88) | 12.16 | 0.002* |
| l-acg | 524 | 0.71 (0.86) | 17.88 | <0.001* |
| r-acg | 623 | 0.85 (1.17) | 13.87 | 0.001* |
| l-stn | 26 | 0.49 (0.69) | 12.93 | 0.001* |
| r-stn | 26 | 0.49 (0.97) | 6.73 | 0.016* |
| b-ver | 359 | 0.52 (1.32) | 3.97 | 0.057 |
| l-put | 232 | 1.04 (1.07) | 24.66 | <0.001* |
| r-put | 227 | 1.06 (1.18) | 20.95 | <0.001* |

ROI, region of interest; SD, standard deviation; l, left; r, right; smg, supramarginal gyrus; ifg, inferior frontal gyrus; pal, pallidum; acg, anterior cingulate gyrus; stn, subthalamic nucleus; ver, vermis; put, putamen. ***** Significant group difference.

**Note.** Brain activity in typically developing children is shown for the expectancy contrast (i.e. all stimuli with expected timing versus all stimuli with unexpected timing).

**Supplementary Table S1b.** Main effect of cognitive control

| ROI | Number of voxels | Control (SD) | F-value  (1,25) | p-value |
| --- | --- | --- | --- | --- |
| l-smg | 1057 | -0.74 (0.78) | 23.24 | <0.001* |
| r-smg | 1015 | -0.91 (0.92) | 24.10 | <0.001* |
| l-ifg | 705 | 0.06 (1.06) | 0.08 | 0.783 |
| r-ifg | 675 | -0.39 (0.98) | 4.02 | 0.056 |
| l-pal | 77 | -0.06 (0.52) | 0.30 | 0.588 |
| r-pal | 75 | -0.20 (0.33) | 9.47 | 0.005* |
| l-acg | 524 | -0.55 (0.61) | 20.67 | <0.001* |
| r-acg | 623 | -0.65 (0.76) | 18.94 | <0.001* |
| l-stn | 26 | 0.02 (0.48) | 12.93 | 0.001* |
| r-stn | 26 | -0.17 (0.47) | 3.50 | 0.073 |
| b-ver | 359 | 0.48 (0.91) | 7.24 | 0.013* |
| l-put | 232 | -0.12 (0.73) | 0.05 | 0.822 |
| r-put | 227 | -0.23 (0.66) | 3.17 | 0.087 |

ROI, region of interest; SD, standard deviation; l, left; r, right; smg, supramarginal gyrus; ifg, inferior frontal gyrus; pal, pallidum; acg, anterior cingulate gyrus; stn, subthalamic nucleus; ver, vermis; put, putamen. ***** Significant group difference.

**Note.** Brain activity in typically developing children is shown for the cognitive control contrast (i.e. all go stimuli versus all nogo stimuli).

**Supplementary Table S2a.** Activity related to timing per region of interest

| ROI | Control (SD) | ADHD (SD) | ASD (SD) | F-value  (2,73) | p-value |
| --- | --- | --- | --- | --- | --- |
| l-smg | 0.92 (1.04) | 0.41 (1.27) | 0.35 (1.54) | 1.49 | 0.232 |
| r-smg | 0.69 (1.51) | 0.29 (1.41) | 0.23 (1.43) | 0.74 | 0.481 |
| l-ifg | 1.00 (1.46) | 0.06 (1.71) | 0.48 (1.11) | 2.68 | 0.075 |
| r-ifg | 0.64 (1.85) | -0.03 (1.47) | 0.35 (1.39) | 1.11 | 0.337 |
| l-pal | 0.65 (0.81) | 0.04 (0.72) | 0.56 (0.59) | 5.36 | 0.007* |
| r-pal | 0.60 (0.88) | 0.08 (0.80) | 0.34 (0.64) | 2.82 | 0.066 |
| l-acg | 0.71 (0.86) | 0.25 (0.78) | 0.33 (0.74) | 2.51 | 0.089 |
| r-acg | 0.85 (1.17) | 0.18 (0.94) | 0.36 (1.00) | 2.85 | 0.064 |
| l-stn | 0.49 (0.69) | -0.23 (0.81) | 0.06 (0.76) | 5.72 | 0.005* |
| r-stn | 0.49 (0.97) | -0.05 (0.88) | 0.14 (0.69) | 2.62 | 0.080 |
| b-ver | 0.52 (1.32) | 0.60 (0.90) | 0.08 (1.27) | 1.38 | 0.259 |
| l-put | 1.04 (1.07) | 0.33 (0.89) | 0.97 (0.77) | 4.54 | 0.014 |
| r-put | 1.06 (1.18) | 0.36 (0.96) | 0.73 (1.04) | 2.69 | 0.080 |

ROI, region of interest; ADHD, Attention-Deficit/Hyperactivity Disorder; ASD, Autism Spectrum Disorder; SD, standard deviation; l, left; r, right; smg, supramarginal gyrus; ifg, inferior frontal gyrus; pal, pallidum; acg, anterior cingulate gyrus; stn, subthalamic nucleus; ver, vermis; put, putamen. ***** Significant overall group difference. ** Significant post-hoc group difference from controls, for post-hoc statistics see Results section.

**Supplementary Table S2b.** Activity related to cognitive control per region of interest

| ROI | Control (SD) | ADHD (SD) | ASD (SD) | F-value  (2,73) | p-value |
| --- | --- | --- | --- | --- | --- |
| l-smg | -0.74 (0.78) | -0.07 (1.14) | -0.33 (0.99) | 3.03 | 0.054 |
| r-smg | -0.91 (0.92) | -0.39 (1.32) | -0.59 (1.26) | 1.18 | 0.313 |
| l-ifg | 0.06 (1.06) | 0.27 (2.21) | -0.15 (1.33) | 0.44 | 0.649 |
| r-ifg | -0.39 (0.98) | -0.17 (1.68) | -0.55 (1.17) | 0.55 | 0.580 |
| l-pal | -0.06 (0.52) | 0.12 (0.83) | 0.12 (0.50) | 0.67 | 0.516 |
| r-pal | -0.20 (0.33) | 0.12 (0.61) | -0.07 (0.56) | 2.38 | 0.099 |
| l-acg | -0.55 (0.61) | -0.16 (0.96) | -0.20 (0.64) | 2.13 | 0.126 |
| r-acg | -0.65 (0.76) | -0.23 (1.05) | -0.30 (0.82) | 1.66 | 0.197 |
| l-stn | 0.02 (0.48) | 0.05 (0.82) | -0.17 (0.44) | 1.01 | 0.369 |
| r-stn | -0.17 (0.47) | -0.06 (0.73) | -0.15 (0.77) | 0.21 | 0.809 |
| b-ver | 0.48 (0.91) | 0.56 (1.64) | 0.55 (1.27) | 0.03 | 0.971 |
| l-put | -0.12 (0.73) | 0.02 (1.11) | 0.18 (0.71) | 0.74 | 0.480 |
| r-put | -0.23 (0.66) | -0.00 (1.00) | -0.08 (0.67) | 0.56 | 0.576 |

ROI, region of interest; ADHD, Attention-Deficit/Hyperactivity Disorder; ASD, Autism Spectrum Disorder; SD, standard deviation; l, left; r, right; smg, supramarginal gyrus; ifg, inferior frontal gyrus; pal, pallidum; acg, anterior cingulate gyrus; stn, subthalamic nucleus; ver, vermis; put, putamen.

**Supplementary Table S3.** Activation differences with performance covariates

| ROI | Factor | β | F-value | p-value |
| --- | --- | --- | --- | --- |
| l-pal | RT_benefit_ | 0.80 | 13.64 | <0.001* |
|  | Group |  | 8.03 | 0.001* |
| l-put | RT_benefit_ | 0.68 | 5.35 | 0.024* |
|  | Group |  | 5.67 | 0.005* |
| l-stn | RT_benefit_ | 0.06 | 0.06 | 0.810 |
|  | Group |  | 5.47 | 0.006* |

ROI, region of interest; l, left; r, right; pal, pallidum; put, putamen; stn, subthalamic nucleus; RT_benefit,_ response time benefit. ***** Significant group difference.

**Supplementary Table S4.** Whole brain activity related to temporal expectancy in typically developing children

| Cl | MNI-x | MNI-y | MNI-z | P-value* | N-vox | Z-value | L/R | Label |
| --- | --- | --- | --- | --- | --- | --- | --- | --- |
| 1 | 24 | -54 | -3 | <0.001 | 981 | 7.12 | R | Lingual gyrus |
| 1 | 15 | -66 | 9 | <0.001 |  | 6.79 | R | Calcerine sulcus |
| 1 | -18 | -66 | 3 | <0.001 |  | 6.44 | L | Calcerine sulcus |
| 2 | -45 | -15 | 45 | <0.001 | 45 | 5.77 | L | Post Central gyrus |
| 3 | -36 | -15 | 6 | 0.001 | 53 | 5.6 | L | Insula |
| 3 | -36 | -18 | 18 | 0.012 |  | 5.08 | L | Insula |
| 3 | -42 | -12 | 15 | 0.044 |  | 4.84 | L | Rolandic operculum |
| 4 | 63 | 0 | 12 | 0.002 | 11 | 5.43 | R | Rolandic operculum |
| 5 | -45 | 0 | 9 | 0.002 | 12 | 5.39 | L | Rolandic operculum |
| 6 | -21 | -87 | 24 | 0.019 | 8 | 5 | L | Superior occipital gyrus |
| 7 | -42 | -33 | 21 | 0.02 | 6 | 4.99 | L | Rolandic operculum |

Cl, cluster; MNI, Montreal Neurological Institute coordinate ; N-vox, number of voxels per cluster; L/R, left/right. * P-value for peak activity corrected for multiple comparisons using family wise error correction (FWE).

**Note.** This table shows brain activity in 26 typically developing children for the expected versus unexpected timing contrast using a one-sided t-test. Only peak activity that reached significance after FWE correction with a minimum cluster size of five voxels is reported. Localization is reported using MNI-coordinates; labels were assigned using Automated Anatomical Labeling (AAL).

| Cl | MNI-x | MNI-y | MNI-z | P-value* | N-vox | Z-value | L/R | Label |
| --- | --- | --- | --- | --- | --- | --- | --- | --- |
| 1 | -36 | 15 | 6 | <0.001 | 231 | 6.43 | L | Insula |
| 1 | -33 | 21 | -6 | <0.001 |  | 6.27 | L | Insula |
| 1 | -30 | 21 | 6 | <0.001 |  | 6.09 | L | Insula |
| 2 | 39 | 18 | -9 | <0.001 | 199 | 6.17 | R | Insula |
| 2 | 36 | 15 | 6 | <0.001 |  | 6.12 | R | Insula |
| 2 | 27 | 21 | -9 | <0.001 |  | 5.89 | R | Insula |
| 3 | -57 | -51 | 33 | <0.001 | 29 | 6.13 | L | Supramarginal gyrus |
| 4 | 3 | 18 | 51 | <0.001 | 26 | 5.73 | R | Supplementary motor area |
| 5 | -9 | 21 | 36 | 0.002 | 9 | 5.42 | L | Midcingulate area |
| 6 | -57 | -39 | 42 | 0.002 | 5 | 5.41 | L | Inferior parietal lobule |
| 7 | -6 | 36 | 18 | 0.005 | 44 | 5.25 | L | Anterior cingulate |
| 7 | 3 | 36 | 27 | 0.006 |  | 5.21 | R | Anterior cingulate |
| 7 | 6 | 39 | 18 | 0.008 | 231 | 5.16 | R | Anterior cingulate |

**Supplementary Table S5.** Whole brain activity related to inhibition in typically developing children

|  |  |  |  |  |  |  |  |  |
| --- | --- | --- | --- | --- | --- | --- | --- | --- |
|  |  |  |  |  |  |  |  |  |
|  |  |  |  |  |  |  |  |  |
|  |  |  |  |  |  |  |  |  |
|  |  |  |  |  |  |  |  |  |
|  |  |  |  |  |  |  |  |  |
|  |  |  |  |  |  |  |  |  |
|  |  |  |  |  |  |  |  |  |
|  |  |  |  |  |  |  |  |  |
|  |  |  |  |  |  |  |  |  |
|  |  |  |  |  |  |  |  |  |
|  |  |  |  |  |  |  |  |  |
|  |  |  |  |  |  |  |  |  |
|  |  |  |  |  |  |  |  |  |

Cl, cluster; MNI, Montreal Neurological Institute coordinate ; N-vox, number of voxels per cluster; L/R, left/right. * P-value for peak activity corrected for multiple comparisons using family wise error correction (FWE).

**Note.** This table shows brain activity in 26 typically developing children for the go versus nogo contrast using a one-sided t-test. Only peak activity that reached significance after FWE correction with a minimum cluster size of five voxels is reported. Localization is reported using MNI-coordinates; labels were assigned using Automated Anatomical Labeling (AAL).
